# Spontaneous Resolution of Massive Spontaneous Tubercular Pneumothorax

**DOI:** 10.1155/2011/502639

**Published:** 2012-01-05

**Authors:** Surya Kant, S. Saheer, G. Hassan, Jabeed Parengal

**Affiliations:** Department of Pulmonary Medicine, Chhatrapati Shahu Ji Maharaj (Erstwhile King George) Medical University, Uttarpradesh Lucknow 226003, India

## Abstract

A 29-year-old female presented with complaints of fever and productive cough of three weeks duration. Pulmonary tuberculosis was diagnosed bacteriologically and she was prescribed antituberculosis drugs. During follow-up she developed massive pneumothorax, for which patient refused surgical management and was managed conservatively. After six months there was complete spontaneous resolution of pneumothorax. The unusual presentation and unexpected outcome prompted us to report this case.

## 1. Introduction

Spontaneous pneumothorax during the course of active pulmonary tuberculosis is a well-known complication with an incidence of 0.6−1.4% [1, 2]. There are published case reports on spontaneous resolution of traumatic pneumothorax with conservative management [3, 4]. Here we present one that may be the first case of spontaneous resolution of massive pneumothorax secondary to tuberculosis. 

## 2. Case Report

**Figure 1:**
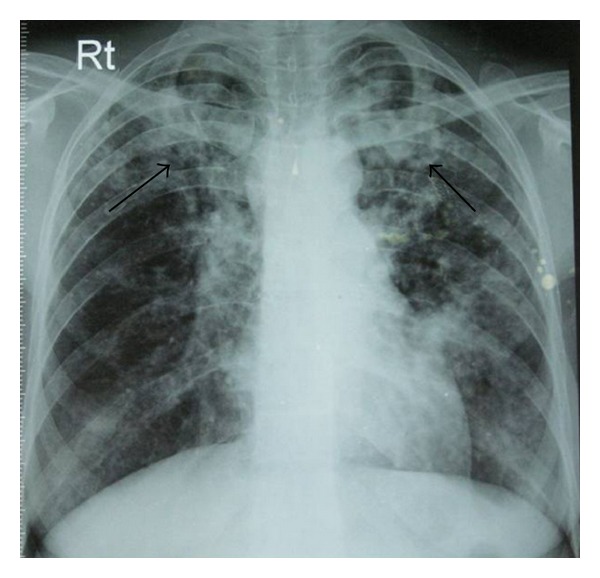
Initial chest radiograph showing bilateral infiltrates (arrows).

**Figure 2:**
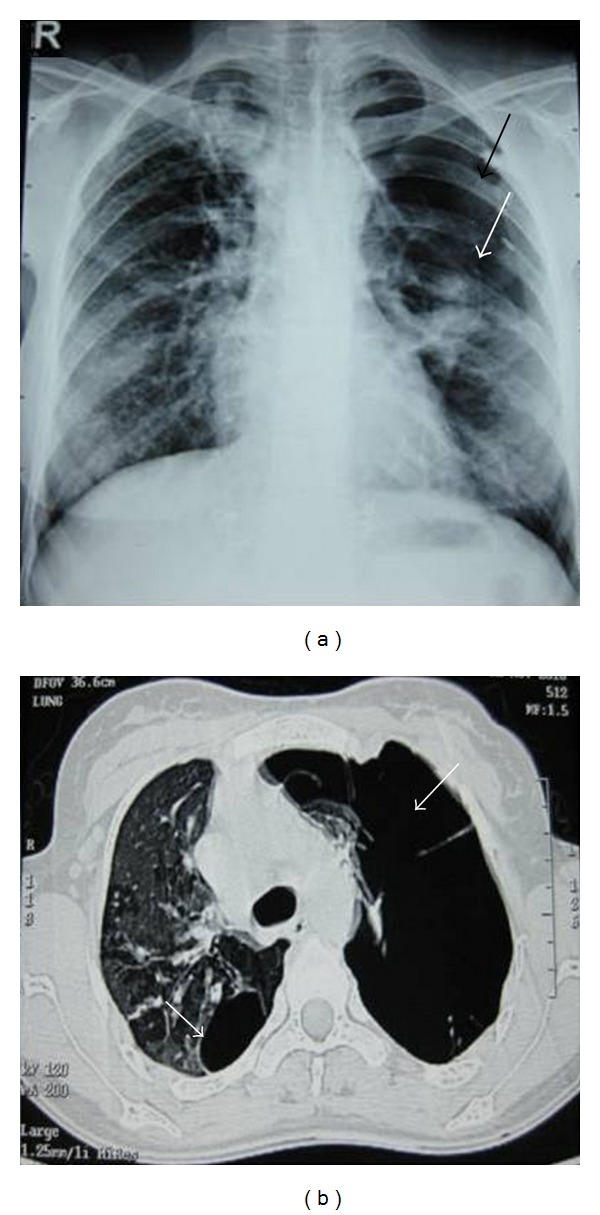
Chest X-ray (a) in the Emergency Department showing pneumothorax of left side (black arrow) with collapsed visceral pleural line (white arrow) and corresponding computed tomography of thorax (b) showing massive pneumothorax of left side (white arrow) with infiltrates and cystic space (white arrow) in right upper lobe.

**Figure 3:**
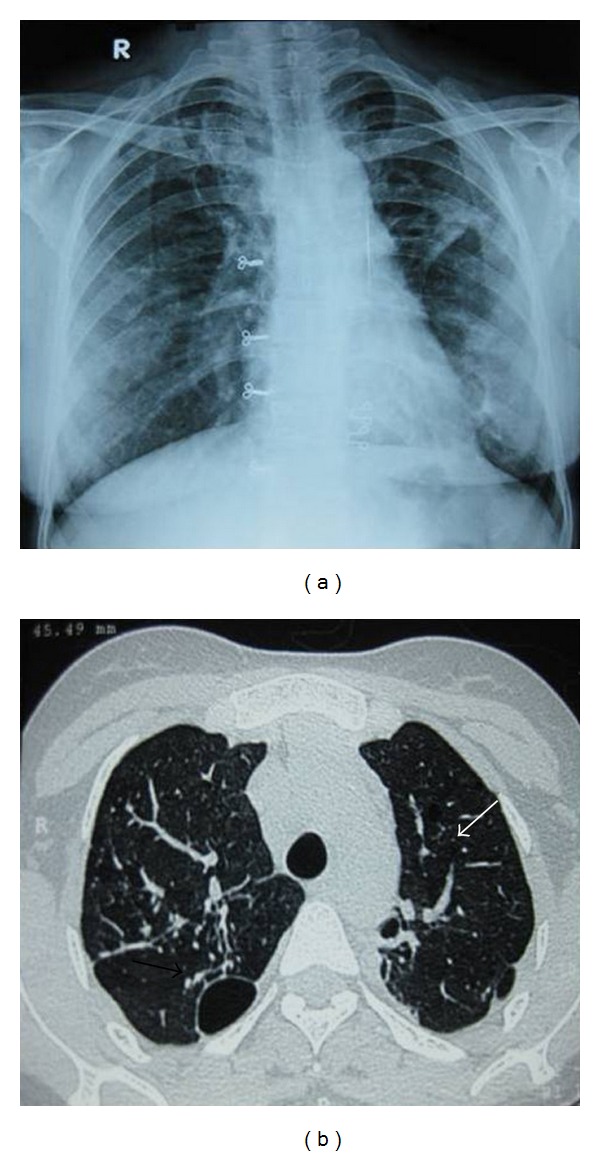
Follow-up (at end of chemotherapy) chest X-ray (a) showing complete resolution of pneumothorax on left side and computed tomography (b) revealing persistence of cystic space in right upper lobe (black arrow) and resolution of left pneumothorax (white arrow).

A 29-year-old nonsmoker lady presented to our department with complaints of fever and productive cough of three-week duration. Her chest radiograph showed bilateral infiltrates more so in upper zones (Figure 1). She had normal haematological and biochemical parameters. There was no clinical evidence of any immunosupression. Acid fast bacilli were found in sputum smears and subsequently culture demonstrated *Mycobacterium tuberculosis*. She was advised of category 1 regimen of the Revised National Tuberculosis Control Programme of India which consists of isoniazid, rifampicin, ethambutol, and pyrazinamide three times weekly for 2 months followed by isoniazid and rifampicin three times weekly for 4 months, but she refused the same and was prescribed daily regimen consisting of isoniazid (300 mg), rifampicin (450 mg), ethambutol (600 mg), and pyrazinamide (1250 mg) for two months followed by isoniazid (300 mg) and rifampicin (450 mg) for four months. After three months of chemotherapy she was readmitted in our emergency department with complaints of left-sided pleuritic chest pain and exertional breathlessness. Her chest radiograph showed pneumothorax on left side (Figure 2(a)) and corresponding computed tomography of thorax (CT) showed massive pneumothorax of left side with infiltrates and cystic spaces in right upper lobe (Figure 2(b)). The patient was advised of intercostal tube drainage but she refused. So she was managed conservatively with oxygen, cough suppressants, and bed rest. Since the patient was smear and culture positive for *Mycobacterium tuberculosis*, both were repeated and the results were negative. With supportive care, her condition improved and was discharged with the advice to continue antituberculosis drugs and to be on regular follow-up. Chest X-ray and CT at the completion of six months of chemotherapy regimen showed complete resolution of pneumothorax (Figures 3(a) and 3(b)). The patient is under our regular follow-up without any complications.

## 3. Discussion

Spontaneous pneumothorax usually occurs in the young individuals without any associated pulmonary or systemic disease. It is usually produced as a result of rupture of subpleural bleb [5]. Secondary spontaneous pneumothorax is associated with significant lung disease either clinical or radiological, and most commonly it occurs secondary to chronic obstructive pulmonary disease and tuberculosis. Overall, around 1% of patients with active tuberculosis present with secondary spontaneous pneumothorax [6]. It occurs as a result of caseous necrosis with bronchopleural fistula formation or rupture of a cavity into the pleural space [5].

Tuberculosis may lead to pneumothorax by several mechanisms and the etiology of pneumothorax in this case remains unknown as there was no visible radiological consolidation or cavity. Probably it may have occurred as a result of rupture of tubercular pneumatocele as also previously reported by Duttaroy et al. [7]. As secondary spontaneous pneumothorax occurs in patients with underlying lung disease, their occurrence is less tolerated by patients. The management protocol includes high flow oxygen, needle aspiration in patients with small pneumothorax, and insertion of small bore chest drain [8]. Our case was exceptional in this aspect that even with massive pneumothorax patient got complete resolution. The patients with pneumothorax secondary to tuberculosis usually have a favourable response to chemotherapy and surgical drainage without relapse of pneumothorax [9]. This observation is expected to alleviate apprehension of physicians while encountering such cases in the usual clinical practice.

## References

[1] Blanco-Pérez J, Bordón J, Piñeiro-Amigo L, Roca-Serrano R, Izquierdo R, Abal-Arca J (1998). Pneumothorax in active pulmonary tuberculosis: resurgence of an old complication?. *Respiratory Medicine*.

[2] Belmonte R, Crowe HM (1995). Pneumothorax in patients with pulmonary tuberculosis. *Clinical Infectious Diseases*.

[3] Karayiannakis AJ, Anagnostoulis S, Michailidis K, Vogiatzaki T, Polychronidis A, Simopoulos C (2005). Spontaneous resolution of massive right-sided pneumothorax occurring during laparoscopic cholecystectomy. *Surgical Laparoscopy, Endoscopy and Percutaneous Techniques*.

[4] Hawasli A (2002). Spontaneous resolution of massive laparoscopy-associated pneumothorax: the case of the bulging diaphragm and review of the literature. *Journal of Laparoendoscopic and Advanced Surgical Techniques: Part A*.

[5] Wilder RJ, Beacham EG, Ravitch MM (1962). Spontaneous pneumothorax complicating cavitary tuberculosis. *The Journal of Thoracic and Cardiovascular Surgery*.

[6] Noppen M, de Keukeleire T (2008). Pneumothorax. *Respiration*.

[7] Duttaroy DD, Jagtap J, Bansal U, Duttaroy B (2006). Tuberculous pulmonary pneumatocele communicating extrathoracically. *Thorax*.

[8] MacDuff A, Arnold A, Harvey J (2010). Management of spontaneous pneumothorax: British Thoracic Society pleural disease guideline 2010. *Thorax*.

[9] Sahn SA, Heffner JE (2000). Spontaneous pneumothorax. *New England Journal of Medicine*.

